# Recombinase polymerase amplification combined with a lateral flow dipstick for rapid and visual detection of *Schistosoma japonicum*

**DOI:** 10.1186/s13071-016-1745-5

**Published:** 2016-08-31

**Authors:** Kui Sun, Weiwei Xing, Xinling Yu, Wenliang Fu, Yuanyuan Wang, Minji Zou, Zhihong Luo, Donggang Xu

**Affiliations:** 1 Beijing Institute of Basic Medical Sciences, Beijing, People’s Republic of China; 2 The key laboratory of Immune and Control of Schistosomiasis, Hunan Institute of Parasitic Diseases, Hunan, People’s Republic of China

**Keywords:** *Schistosoma japonicum*, Recombinase polymerase amplification, Lateral flow dipstick, Visual detection, Field application

## Abstract

**Background:**

With the continuous decline in prevalence and intensity of *Schistosoma japonicum* infection in China, more accurate and sensitive methods suitable for field detection become much needed for schistosomiasis control. Here, a novel rapid and visual detection method based on the combination of recombinase polymerase amplification (RPA) and lateral flow dipstick (LFD) was developed to detect *S. japonicum* DNA in fecal samples.

**Results:**

The LFD-RPA assay targeting SjR2 could detect 5 fg *S. japonicum* DNA, which was identical to qPCR and real-time RPA assay, and showed no cross-reaction with other parasites. The detection could be finished within 15–20 min at a wide temperature range (25–45 °C), and the results could be visualized by naked eye. The diagnostic validity of LFD-RPA assay was further assessed with 14 fecal samples of infected patients diagnosed by Kato-Katz method and 31 fecal samples of healthy persons, and compared with that of Enzyme-linked immunosorbent assay (ELSIA) and Indirect Hemagglutination Assay (IHA). The LFD-RPA assay showed 92.68 % sensitivity, 100 % specificity and excellent diagnostic agreement with the gold standard Kato-Katz test (*k* = 0.947, *Z* = 6.36, *P* < 0.001), whereas ELISA showed 85.71 % sensitivity, 93.55 % specificity, and substantial diagnostic agreement (*k* = 0.793, *Z* = 5.31, *P* < 0.001), and IHA showed 78.57 % sensitivity, 83.87 % specificity, and moderate diagnostic agreement (*k* = 0.600, *Z* = 4.05, *P* < 0.001), indicating that the LFD-RPA was much better than the traditional methods.

**Conclusions:**

The LFD-RPA assay established by us is a sensitive, specific, rapid and convenient method for the diagnosis of schistosomiasis, and shows a great potency in field application.

**Electronic supplementary material:**

The online version of this article (doi:10.1186/s13071-016-1745-5) contains supplementary material, which is available to authorized users.

## Background

Schistosomiasis is one of the oldest parasitic infestations of humankind with a documented history of > 2,100 years in China [1]. Since the foundation of the People’s Republic of China, the government has placed a high priority on the control of schistosomiasis and the ongoing national control program has made great progress in controlling this disease [2, 3]. Results showed that, by the end of 2012, the estimated number of people infected with *Schistosoma japonicum* (*S. japonicum*) had decreased from approximately 11.6 million cases in the mid-1950s to 240,597 cases; meanwhile, the number of endemic provinces had been reduced from 12 to 7 [4]. In spite of these achievements, the Ministry of health reported that progress on schistosomiasis control has been rather slow in recent years [4]. One important factor contributing to the situation is the scarcity of effective diagnostic methods for surveillance of low-intensity infections and assessment of intervention effectiveness [5]. Conventional diagnostic methods, including direct parasitological and immunological techniques, are not sufficiently sensitive to accurately determine the prevalence of schistosomiasis or parasite burden [6]. Direct parasitological examinations, such as the Kato-Katz thick smear (KK) and the miracidium hatching test (MHT), are the most extensively used techniques in field surveys because they exhibit high specificity and are relatively inexpensive. However, they are time consuming (KK requires 4 to 5 h and MHT needs more than 6 h) and relatively insensitive, and thus cannot satisfy the requirements of low-prevalence areas of *S. japonicum* in China [7–9]. Indirect immunological techniques including IHA and ELISA are extensively employed methods for community diagnosis and screening of people targeted for chemotherapy, owing to its relatively high sensitivity, simplicity and rapidity in field detection compared with the KK and MHT methods. However, they cannot distinguish active infection from previous infection and have a high cross-reactivity with other parasites, which disturb its continued wide application in China [10, 11]. In addition, some molecular assays, such as the PCR-based methods, are capable of detecting *S. japonicum* DNA in a variety of samples [12]. Although studies demonstrate that they are sensitive and reliable, these PCR techniques are restricted to the laboratory due to the requirements for instrumentation and specific conditions. Therefore, sensitive, specific, rapid and convenient diagnostic methods for field setting are much needed to achieve the final elimination of schistosomiasis.

RPA is a novel isothermal technique that does not require an initial heating step to denature DNA template, but employs recombinase-primer complexes to scan double-stranded DNA and facilitate strand exchange at cognate sites [13]. It does not require precise temperature control and can amplify DNA to detectable levels in less than 20 min [14, 15]. Lateral flow dipstick (LFD) is a simple device currently used for qualitative, semi-quantitative and to some extent quantitative monitoring in resource-poor or non-laboratory environments [16]. The combination of RPA and LFD can establish a sensitive, specific, quick and visual detection system for trace target species in the field.

In this study, we developed a LFD-RPA assay for detecting *S. japonicum* DNA in fecal samples and assessed its diagnostic validity. The sensitivity of LFD-RPA assay was evaluated by comparison with that of qPCR and real-time RPA methods. The effectiveness of LFD-RPA assay was determined with clinical stool samples and the agreement between LFD-RPA assay and the gold standard Kato-Katz test was calculated using Kappa test.

## Methods

### Study population and sample collection

Fourteen patients from Xiangyue Hospital diagnosed with schistosomiasis by the Kato-Katz (KK) method and 31 healthy volunteers without schistosomiasis history living in Haidian District of Beijing, where schistosomiasis is not endemic, participated in the study. Written informed consent was obtained from all adult participants and from parents or legal guardians of minors. The fecal samples were first evaluated for the presence of *S. japonicum* eggs by the Kato-Katz method and subsequently stored at -70 °C until DNA extraction. The serum samples were collected and stored at -70 °C for the IHA and ELISA test.

### DNA extraction

Genomic DNA extracted from adult *S. japonicum* worms provided by Hunan Institute of Parasitic Diseases was used to prepare a DNA standard for sensitivity analysis. Total DNA of stool samples was extracted using the QIAamp DNA Stool Mini Kit (Qiagen GmbH, Hilden, Germany) according to the manufacturer’s protocols. Quantity of the total DNA obtained from 200 mg fecal samples usually ranged between 2.5 and 3.9 μg/50 μl.

### Primer and probe design

The highly repetitive retrotransposon SjR2 of *S. japonicum* (GenBank accession No. AF412221) was used for DNA detection as a target sequence [17–19]. The SjR2-specific primers for LFD-RPA and real-time RPA were designed according to Piepenburg [13]. Their primer sequences were identical except for an additional biotin-label at the 5′ end of the reverse primer of LFD-RPA. Several primer combinations were evaluated and one primer pair was eventually identified. PCR primers were designed with the Primer 3.0 [20]. Primers and probes for LFD-RPA, real-time RPA and qPCR are listed in Table 1. All oligonucleotides were produced by Sangon Biotech, Beijing, China.

**Table 1:** List of primers and probe for the lateral-flow stripe, real-time RPA assay and qPCR based on the *Schistosoma japonicum* genomic DNA

Assay format	Name	Sequence 5′–3′
Lateral-flow stripe RPA	RF	CCCAAGTCTCAGTGAAGTTGTGAAGGCTAT
	RR	Biotin-GTTAGTGTTCGAGACCAGTCAGATGGGATT
	Probe	FAM-CTTAAAGCGAGGGAGAGCGGCAGGACCAG
		ATG[THF]ATTGACCCCTGAGATAT-ph
Real- time RPA	RF	CCCAAGTCTCAGTGAAGTTGTGAAGGCTAT
	RR	GTTAGTGTTCGAGACCAGTCAGATGGGATT
	Probe	CTTAAAGCGAGGGAGAGCGGCAGGACCAGA
		[FAM-dT]G[THF]A[BHQ-dT]TGACCCCTGAGATAT-/C3-spacer/
qPCR	RF	GACAGGTTCTGGAACATAGG
	RR	GGTCAATTCCGAAGACAATC

*Abbreviations*: *FAM* 6-Carboxyfluorescein, *THF* tetrahydrofuran, *BHQ* black hole quencher

### Real-time RPA assay

Briefly, each reaction contained 29.5 μl of rehydration solution, 10.7 μl of nuclease-free water, 2.1 μl of each primer (10 mM), 0.6 μl of the RPA probe (10 mM) and 2.5 μl of template with a total of 0, 1, 5, 10, 10^2^, 10^3^, 10^4^, 10^5^ fg *S. japonicum* genomic DNA standard were added to each reaction. To initiate the reaction 2.5 μl of magnesium acetate was added. Reactions were performed at 39 °C in a real-time fluorometer (Twista®, TwistDx) for 20 min with a mixing and centrifugation step after the first 4 min [21, 22].

### LFD-RPA assay

A typical 50 μl reaction was performed with the TwistAmp Exo® kit. For each reaction, 29.5 μl of rehydration buffer, 0.6 μl of TwistAmp LF probe which contained a 5′ FAM, an internal abasic site (tetrahydrofuran) and a 3′ polymerase extension blocking group (10 μM), 2.1 μl of unlabeled forward primer (10 μM), 2.1 μl of biotin-labeled reverse primer (10 μM), 10.7 μl of nuclease-free water, and 2.5 μl of *S. japonicum* genomic DNA standard or total DNA extracted from clinical stool samples. Finally, 2.5 μl of magnesium acetate was added to the system to generate a RPA amplicon labeled with FAM and biotin, which could be detected on the lateral flow strips.

After amplification, the RPA products were diluted 10-fold with the provided running buffer. Commercially available HybriDetect lateral flow strips (Milenia Biotec, Germany) were vertically inserted into the dilution and incubated for 5 min. Pictures were then taken by a Sony camera (NEX-5).

### qPCR assay

The qPCR assay was performed in a 20 μl reaction containing 10 μl of Ultra SYBR Green Mixture (with ROX), 1 μl of reverse primer, 1 μl of forward primer, 7 μl of ddH_2_O, 1 μl of template containing a total of 0, 1, 5, 10, 10^2^, 10^3^, 10^4^, 10^5^ fg *S. japonicum* genomic DNA standard were added to each reaction. The thermal cycler program was as follows: 95 °C for 10 min, followed by 40 cycles of 94 °C for 15 s and 60 °C for 60 s. The assay was performed on an ABI 7,500 with 7,500 software v2.0 (Applied Biosystems, USA).

### Kato-Katz thick smear

The Kato-Katz thick smear was performed with tissue papers, wire meshes and standard templates that can hold 41.7 mg of feces [9]. First, approximately 1 g of feces was placed on a tissue paper and covered with a wire mesh. With the aid of a spatula, feces were strained through the mesh. The fine slag was deposited onto a standard template located on a glass slide. A strip of cellophane paper embedded in 3 % malachite green-glycerol solution was covered on top of the feces after removing the template. Finally, the preparations were kept at room temperature for 30 to 60 min and examined by microscope using 100-fold magnification. The number of *S. japonicum* eggs per gram (epg) of feces was obtained by multiplying the number of eggs per glass slide by 24.

### ELISA and IHA

The ELISA to detect *S. japonicum* antigen-specific IgG was performed as described in the *S. japonicum* IgG ELISA kit (BIOCBD, Shenzhen, China). Serum samples at a dilution of 1:100 and two negative controls provided with the kit were used. A positive antibody test was defined as an OD value greater than 2.1 times the mean value of the negative controls.

IHA was performed by collecting sera in capillary tubes and using a commercially available kit from the Anhui Provincial Institute of Parasitic Diseases (Wuhu, China). An antibody titer of at least 1:10 was recognized as the positive criterion [9].

### Evaluation of LFD-RPA

The sensitivity of the LFD-RPA assay was evaluated using dilutions of *S. japonicum* genomic DNA and compared with real-time RPA and q-PCR. The specificity was confirmed with DNA samples from four non-related species (*Schistosoma sinensium*, S*chistosoma mansoni*, *Echinochasmus japonicus*, *Clonorchis sinensis*). The amplification temperature and time for RPA are two key parameters for its field application. Therefore, various temperatures ranging from 20 to 50 °C were chosen to determine the optimal temperature for the assay. The reactions for the time experiments were performed at 39 °C and stopped by placing the tubes in an ice box at 0, 5, 10, 15, 20 and 25 min.

### Statistical analysis

The stool and serum samples from 14 infected patients diagnosed with K-K and 31 healthy volunteers were tested in parallel with the LFD-RPA, ELISA and IHA assays. Data were stored in an Excel 2003 spreadsheet and analyzed with SAS version 9.2. The diagnostic performance of the LFD-RPA, IHA and ELISA assays was assessed by calculating the sensitivity, specificity, positive predictive value (PPV), negative predictive value (NPV) and 95 % confidence intervals (CIs). Diagnosis agreement of the Kato-Katz method with the other methods was analyzed with the kappa test. The coefficient of *k* was defined as (Po−Pe)/(1 − Pe) and after Landis & Koch [23] the agreement based on characterized values were considered as: excellent (1.00–0.81); substantial (0.80–0.61); moderate (0.60–0.41); weak (0.40–0.21); and negligible (0.20–0).

## Results

### Sensitivity analysis

**Fig. 1:**
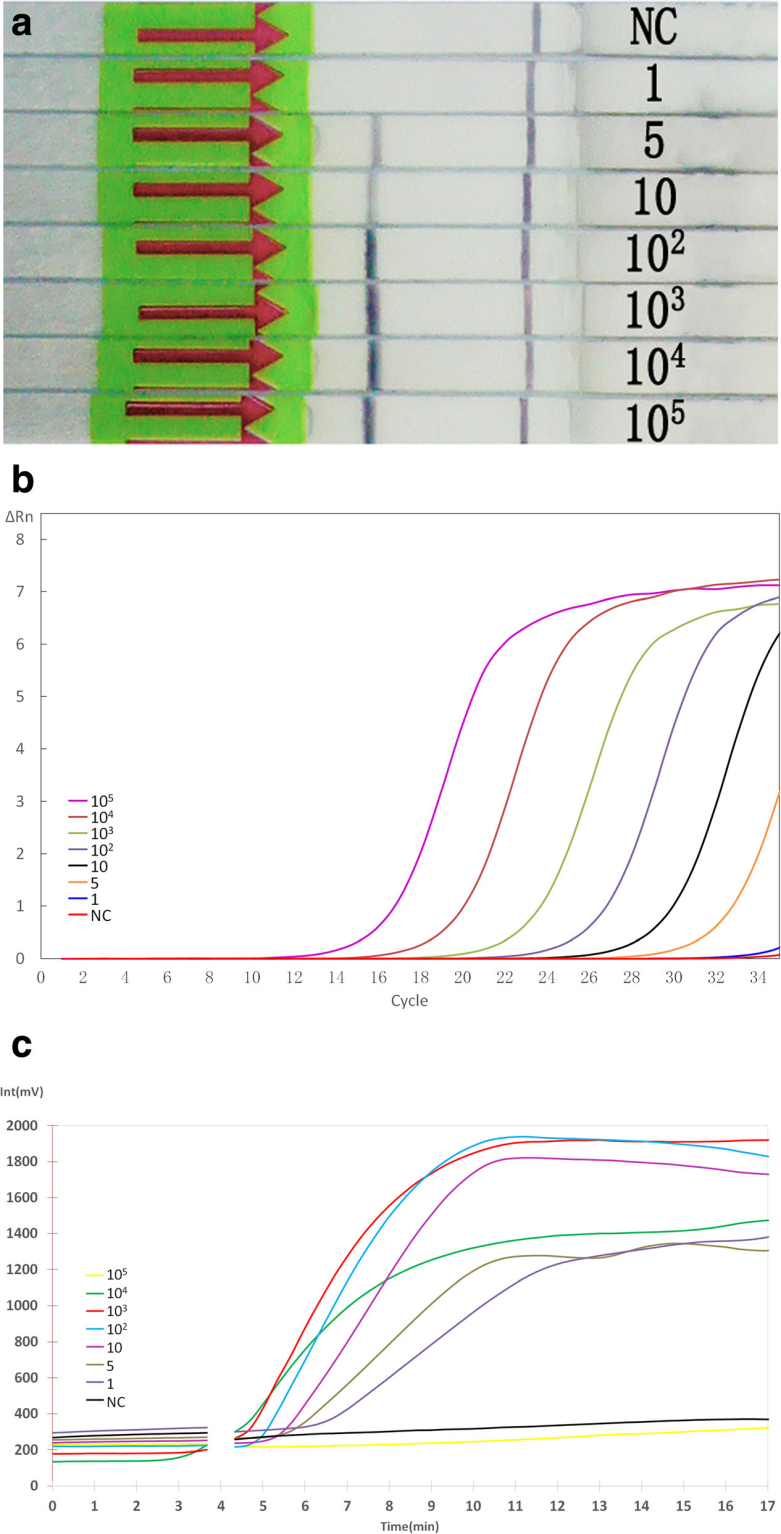
Comparison of the sensitivities of the LFD-RPA, qPCR, and real-time RPA assays. Serial dilutions of adult *S. japonicum* DNA were used to test the analytical sensitivity of these assays. The LFD-RPA assay showed the same sensitivity (5 fg) as qPCR and real-time RPA. **a** Results by LFD-RPA. **b** Results by qPCR. **c** Results by real-time RPA. *Abbreviation*: NC, negative control

The *S. japonicum* genomic DNA from 10^5^ fg to 1 fg by serial dilution was used for threshold detection of the LFD-RPA, real-time RPA and qPCR methods. The results demonstrated that the LFD-RPA was able to detect more than 5 fg DNA (Fig. 1a). As shown, two clear bands on the lateral flow dipsticks were presented from 5 fg to 10^5^ fg genomic DNA, while only the control band could be seen for the 1 fg genomic DNA and negative control reaction. The real-time RPA and qPCR showed the same detection limit as the LFD-RPA (Fig. 1b, c), and the melting curve analysis of qPCR demonstrated good specificity (data not shown).

### Specificity analysis

**Fig. 2:**
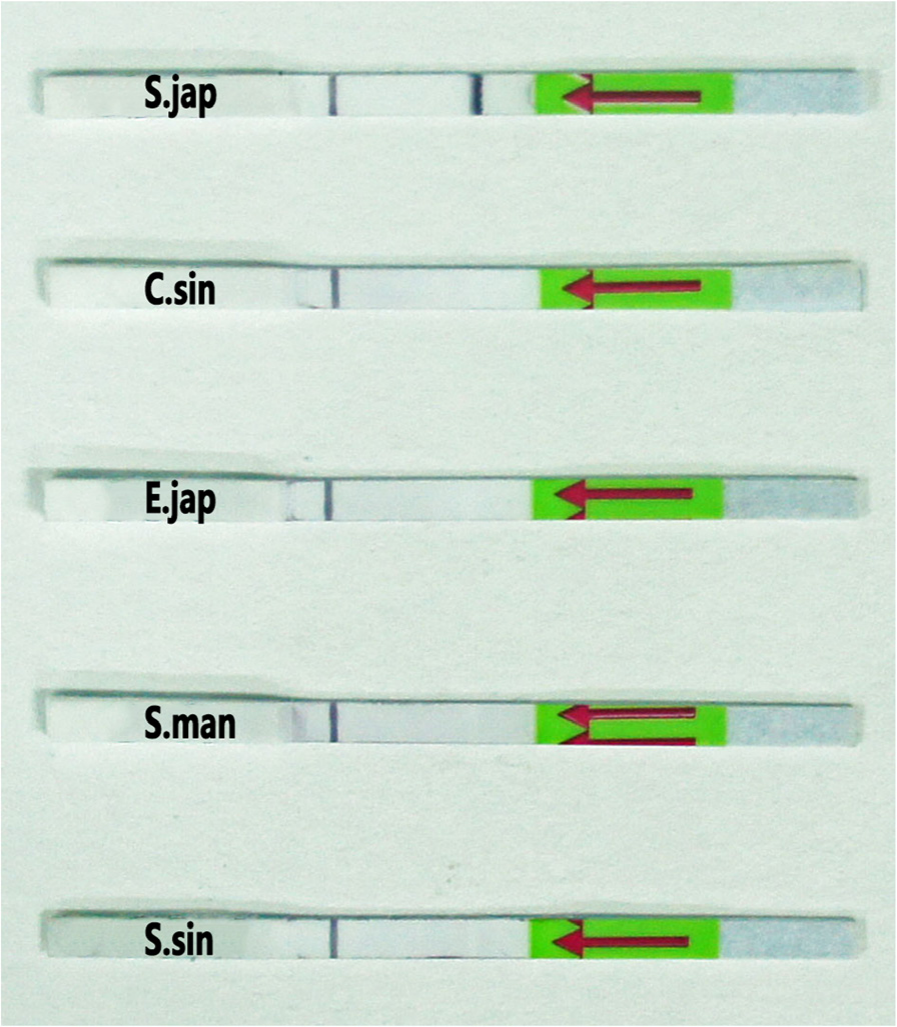
Specificity of the LFD-RPA assay. The analytical specificity test revealed that DNAs from *S. sinensium*, *S. mansoni*, *E. japonicus*, and *C. sinensis* showed no cross-reaction in the developed *S. japonicum*-specific assay

The specificity of the LFD-RPA primers was determined by using four different non-related DNAs from *S. sinensium*, *S. mansoni*, *E. japonicus* and *C. sinensis*. It showed that the LFD-RPA assay had a good ability to discriminate its target DNA from that of other parasites. As shown in Fig. 2, except for *S. japonicum* samples, red-purple color line was only observed as the control line on the LFD strips. These results demonstrated that *S. japonicum* LFD-RPA assay was specific for detection of *S. japonicum*.

### Evaluation of amplification temperature

**Fig. 3:**
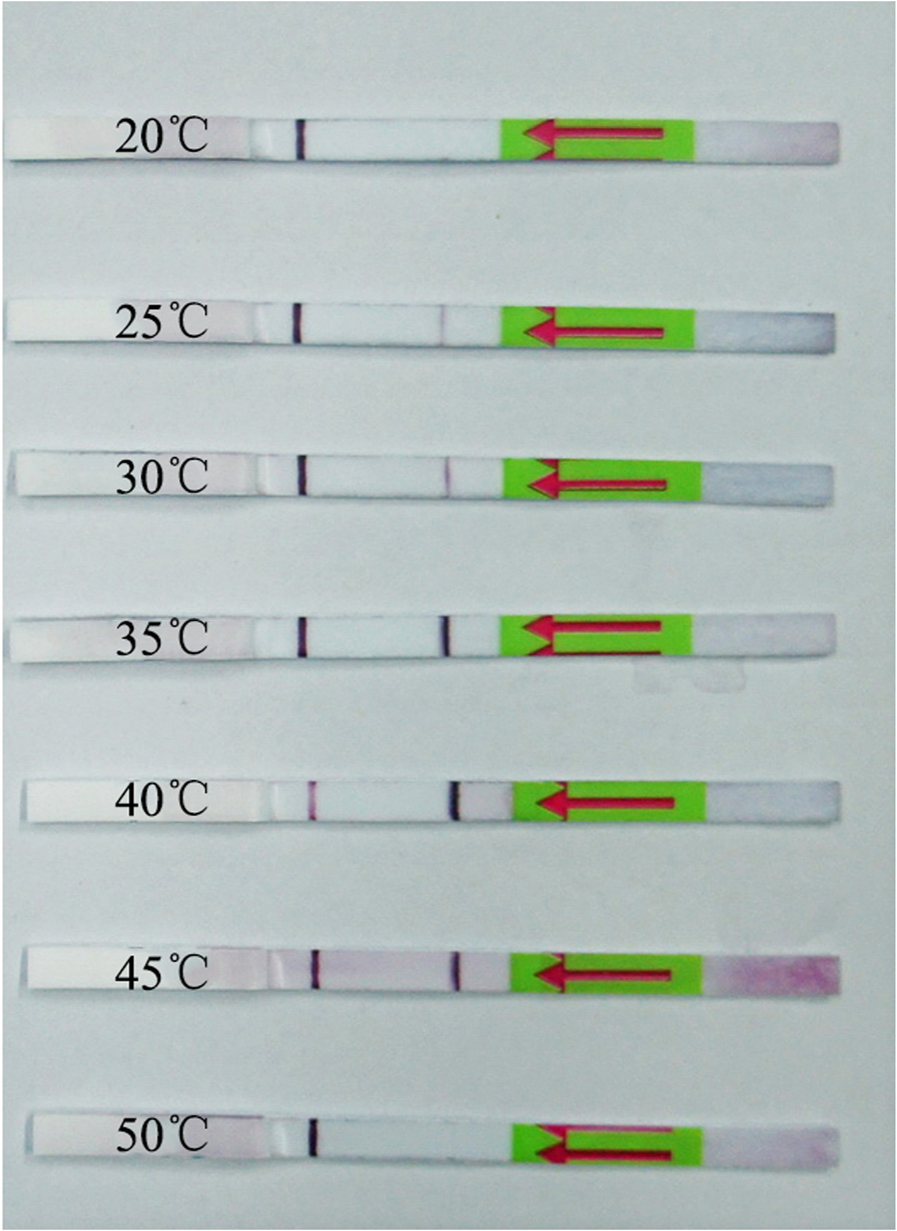
Evaluation of amplification temperature. Lateral flow strip results were positive for reactions performed in temperatures between 25–45 °C, suggesting that the assay works effectively in a broad range of constant reaction temperatures

The results showed that the assay could be performed at a wide range of temperatures from 25 to 45 °C (Fig. 3). The brightness of the test bands appeared to change with temperature, indicating that temperature was critical for amplification efficiency. A clearly visible test band on the dipstick appeared within the temperature range 35–45 °C, and became weaker in brightness as the temperature fluctuated out of the range.

### Evaluation of amplification time

**Fig. 4:**
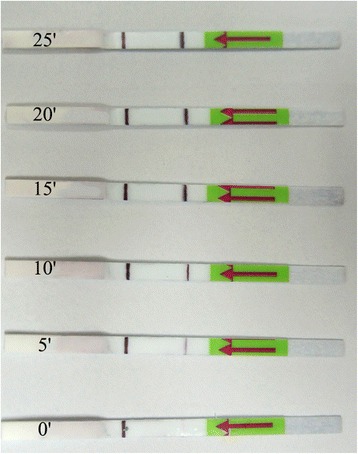
Evaluation of amplification time. After 5 min of amplification reaction, a faint test line was visible on the test strip, suggesting that the amplified target DNA can be detected when the amplification time is less than 10 min

The LFD-RPA reaction time is divided into RPA amplification time and LFD incubation time. The RPA amplification was conducted at 39 °C for 5–25 min while the LFD incubation was conducted at room temperature for 5 min. In our preliminary experiment, we found that obvious signal could be seen at 1–2 min when the amplification time was 20 min, therefore, an incubation period of 5 min for the lateral flow was chosen in order to assess all tests evenly. We demonstrated that a faint test line could be visible when the amplification time was set at 5 min (Fig. 4). As the amplification time increased to 10 min, performance improved with a strong positive signal at the test line. To provide maximum sensitivity while keeping assay rapid for use at the point-of-care, we recommend an amplification time of 10–15 min for future experiments, despite a 5-min amplification time was sufficient in this experiment.

### Analysis of clinical samples

**Fig. 5:**
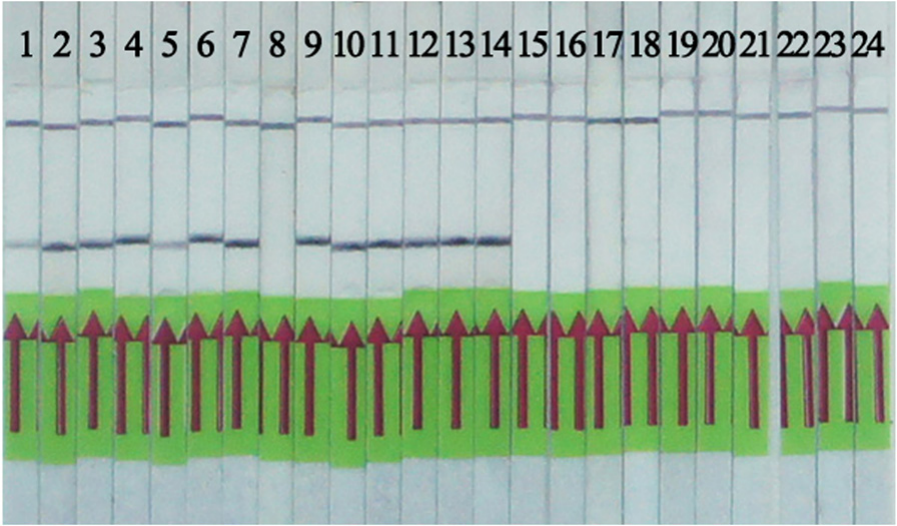
Detection of clinical samples with the LFD-RPA assay. The amplification product can be visualized using lateral flow strips. Of the 14 confirmed samples, 13 yielded a positive result while all the 31 samples from health volunteers were negative by LFD-RPA assay. Some of the negative results were not displayed

The stool samples from 14 confirmed patients and 31 healthy volunteers were examined by LFD-RPA, ELISA and IHA. Diagnostic parameters, including sensitivity, specificity, positive predictive value (PPV), negative predictive value (NPV), and 95 % confidence intervals (CIs), were used to evaluate their diagnostic validity. As shown in Fig. 5, of the 14 fecal samples from confirmed patients, 13 yielded a positive result by the LFD-RPA assay, and the other 31 samples from healthy volunteers showed negative results (data not shown). Table 2 and Additional file 1: Table S1 show that all of the parameters of the LFD-RPA assay were superior than those of the other tested methods. The sensitivity of the LFD-RPA was 92.68 %, whereas those of the IHA and ELISA assays were 78.57 and 85.71 %, respectively. The specificity of the LFD-RPA assay was 100 %, whereas those of the IHA and ELISA assays were 83.87 and 93.55 %, respectively.

**Table 2:** Sensitivity, specificity and predictive value of LFD-RPA, IHA and ELISA assays for diagnosing *S. japonicum* infection

Method	Sensitivity	Specificity	PPV	NPV
LFD-RPA	0.9268	1.000	1.000	0.9688
IHA	0.7857	0.8387	0.6875	0.897
ELISA	0.8571	0.9355	0.857	0.934

*Abbreviations*: *PPV* positive predictive value, *NPV* negative predictive value

The strength of agreement between KK method and the other three detection methods was determined with the kappa test (SAS version 9.2). Calculation of the agreement between the LFD-RPA and Kato-Katz test resulted in a value of *k* = 0.947, *Z* = 6.36, *P* < 0.001, whereas the corresponding values for the ELISA and IHA were *k* = 0.793, Z = 5.31, *P* < 0.001 and k = 0.600, Z = 4.05, *P* < 0.001 respectively (Table 3). Therefore, we conclude that LFD-RPA had good agreement with the Kato-Katz test, whereas ELISA and IHA had moderate agreement.

**Table 3:** Kappa values of LFD-RPA, IHA and ELISA assays on clinical samples

Method	Kappa value	*Z*	*P*
LFD-RPA	0.947	6.36	< 0.001
IHA	0.600	4.05	< 0.001
ELISA	0.793	5.31	< 0.001

## Discussion

Nowadays, schistosomiasis decreases significantly in prevalence and intensity of infection in China. However, the current diagnosis methods are either inadequately sensitive or inconvenient, which severely limit their application, so more accurate and sensitive methods for field diagnosis are much needed for its further control [24–27]. RPA is a relatively new isothermal amplification method that can amplify target DNA to detectable levels in less time and at lower temperatures than that of other isothermal amplification techniques [28–30]. In addition, RPA is tolerant to impure samples [31] and its reagents are available in a lyophilized form that can be transported without requiring cold chain storage. The above advantages make it particularly suitable for field diagnosis. A lateral flow strip is a visual testing tool that eliminates the need for trained personnel and expensive equipment. Therefore, the platform composed by RPA and lateral flow strip shows impressive advantages, such as high sensitivity, good specificity, convenient operation, rapid reaction and less energy requirement, which make it perfect for field diagnosis of *S. japonicum* infection [32, 33].

Here, the *S. japonicum* LFD-RPA system was established and its diagnostic validity was evaluated in healthy and infected people, respectively. The sensitivity of this method was found to be identical to that of real-time RPA and qPCR, and it could detect 5 fg of *S. japonicum* genomic DNA, which was equivalent to less than DNA in a single egg [34]. Regarding the specificity of the LFD-RPA system, the primers were designed targeted SjR2 DNA of *S. japonicum*, which was identified as a DNA marker for effective diagnosis of *S. japonicum* infection, and there was no cross-reaction with other parasite infections [17–19]. The above results demonstrated that the *S. japonicum* LFD-RPA system had high sensitivity and good specificity in the detection of *S. japonicum* infection.

The additional advantage of the *S. japonicum* LFD-RPA system was the shorter incubation time and lower, single incubation temperature. Our study showed that the LFD-RPA assay could amplify target DNA at a relatively low, constant temperature from 25 to 45 °C, whereas the optimum reaction temperature was 35 to 45 °C. It means that a simple heating device, such as a battery-powered heater, chemical heater, or even body heat, can be used to achieve accurate detection [14, 15]. Meanwhile, the LFD-RPA assay could amplify the target DNA to detectable levels within 5–10 min, which was much shorter than that of other detection methods such as PCR, microscopy, LAMP and so on. In addition, LFD-RPA results were presented as reddish band on the lateral flow dipsticks which could be read with a naked eye by untrained personnel. This is especially important for epidemic areas where instruments and trained healthcare workers are deficient.

Subsequently, further tests were conducted to evaluate the diagnostic validation of the *S. japonicum* LFD-RPA assay with clinical samples and compare it with IHA and ELISA, which are widely applied in field studies. Here we chose stool as the clinical sample which was easily acquired and non-invasive. The LFD-RPA assay showed 92.9 % sensitivity and 100 % specificity, which were much higher than that of IHA and ELISA. Previous studies showed that the false-positive rates of the IHA and ELISA were very high as they could not distinguish current infection from past, and easily cross-reacted with antibodies of other parasites [10, 11]. In addition, the kappa test demonstrated that the assay had a significantly higher degree of agreement with the gold standard than that of IHA and ELISA.

Overall, LFD-RPA is an attractive method for the accurate and rapid diagnosis of schistosomiasis, which is suitable not only for point-of-care diagnosis but also for field screening and mass monitoring of the disease. However, before the LFD-RPA assay can be applied in clinical practice for the detection of *S. japonicum*, further improvements are required. One drawback of the current assay is that the LFD-RPA assay is unsealed during detection, which might cause false positives in practical applications. Now we are designing and verifying a novel sealed device suitable for amplification and detection of LFD-RPA system that is different from the commercial AmpliVue (QUIDEL, USA), which is still unsealed and easily disturbed by aerosol pollution of nuclear acid amplicon. The other drawback is its high cost (approximately 8 USD per test). Of course, the prices can be further decreased in the future with its increase of availability and throughput. In addition, the DNA extraction method applied in the assay is not suitable for the field application. The LFD-RPA assay should also be combined with a simple DNA extraction method to assemble a sample-to-answer *S. japonicum* DNA detection system. The commercially available magnetic bead-based strategy, non-commercial ROSE extraction method and heated NaOH method are potential tools for the nucleic acid extraction at field detection though they still require further evaluation and optimization [35, 36].

## Conclusion

In summary, we establish a novel LFD-RPA assay for *S. japonicum* detection, which offers significant advantages regarding high sensitivity, good specificity, rapid and visual detection, and the minimal equipment requirement. All of these qualities make the LFD-RPA assay a promising on-site detection technology for monitoring of *S. japonicum* infection.

## Additional files

Additional file 1: Table S1. Assessed the diagnostic validity of LFD-RPA assay with 45 clinical samples and compared with that of IHA and ELISA assays. (DOCX 15 kb)

## Abbreviations

ELISA: Enzyme-linked immunosorbent assay
EPG: Eggs per gram
FAM: Carboxyfluorescein
IHA: Indirect hemagglutination assay
KK: Kato-katz thick smear
LAMP: Loop-mediated amplification
LFD: Lateral flow dipstick
LFD-RPA: Lateral-flow stripe recombinase polymerase amplification
MHT: The miracidium hatching test
RPA: Recombinase polymerase amplification
THF: Tetrahydrofuran abasic-site mimic
