# Supplementary material for: Recombinase polymerase amplification combined with a lateral flow dipstick for rapid and visual detection of Schistosoma japonicum
Source: Parasit Vectors. 2016 Aug 31;9(1):476. doi: 10.1186/s13071-016-1745-5 (PMC5006264; doi:10.1186/s13071-016-1745-5)
Supplement: Additional file 1: Table S1. — Assessed the diagnostic validity of LFD-RPA assay with 45 clinical samples and compared with that of IHA and ELISA assays. (DOCX 15 kb) [file 13071_2016_1745_MOESM1_ESM.docx]

**Table S1. Assessed the diagnostic validity of LFD-RPA assay with 45 clinical samples and compared with that of IHA and ELISA assays.**

| **Method** | **True positive** | **False positive** | **True negtive** | **False negtive** | **Total** |
| --- | --- | --- | --- | --- | --- |
| **LFD-RPA** | 13 | 0 | 31 | 1 | 45 |
| **ELISA** | 12 | 2 | 29 | 2 | 45 |
| **IHA** | 11 | 5 | 26 | 3 | 45 |

| **Method** | **Sensitivity** | **Specificity** | **+LR** | **-LR** | **PPV** | **NPV** |
| --- | --- | --- | --- | --- | --- | --- |
| **LFD-RPA** | 0.9268 | 1 | - | 0.0714 | 1.000 | 0.9688 |
| **ELISA** | 0.8571 | 0.9355 | 13.2857 | 0.1527 | 0.8571 | 0.9355 |
| **IHA** | 0.7857 | 0.8387 | 4.8714 | 0.2555 | 0.6875 | 0.8699 |

+LR is Positive likelihood ratio; -LR, Negative likelihood ratio; PPV, Positive predictive value; NPV, Negtive predictive value.
